# Overexpression of *miR-1* in the heart attenuates hippocampal synaptic vesicle exocytosis by the posttranscriptional regulation of SNAP-25 through the transportation of exosomes

**DOI:** 10.1186/s12964-018-0303-5

**Published:** 2018-11-29

**Authors:** Ming-Jing Duan, Mei-Ling Yan, Qin Wang, Meng Mao, Dan Su, Lin-Lin Sun, Ke-Xin Li, Yang Qu, Qiang Sun, Xin-Yu Zhang, Si-Yu Huang, Ji-Chao Ma, Tao Ban, Jing Ai

**Affiliations:** Department of Pharmacology, College of Pharmacy of Harbin Medical University (the State-Province Key Laboratories of Biomedicine-Pharmaceutics of China), Harbin, 150086 Heilongjiang Province China

**Keywords:** Heart, Hippocampus, Synaptic vesicle exocytosis, *microRNA-1*, SNAP-25

## Abstract

**Background:**

The link between cardiac diseases and cognitive deterioration has been accepted from the concept of “cardiogenic dementia”, which was proposed in the late 1970s. However, the molecular mechanism is unclarified.

**Methods:**

The two animal models used in this study were cardiac-specific overexpression of microRNA-1-2 transgenic (Tg) mice and a myocardial infarction mouse model generated by left coronary artery ligation (LCA). First, we observed the *microRNA-1* (*miR-1*) level and synaptic vesicles (SV) distribution in the hippocampus using in situ hybridization and transmission electron microscopy (TEM) and evaluated the expression of vesicle exocytosis related proteins by western blotting. Second, we used dual luciferase reporter assay as well as antagonist and miRNA-masking techniques to identify the posttranscriptional regulatory effect of *miR-1* on the *Snap*25 gene. Third, FM1–43 staining was performed to investigate the effect of *miR-1* on synaptic vesicle exocytosis. Lastly, we used GW4869 to inhibit the biogenesis and secretion of exosomes to determine the transportation effect of exosomes for *miR-1* from the heart to the brain.

**Results:**

Compared with the levels in age-matched WT mice, *miR-1* levels were increased in both the hearts and hippocampi of Tg mice, accompanied by the redistribution of SVs and the reduction in SV exocytosis-related protein SNAP-25 expression. In vitro studies showed that SNAP-25 protein expression was down- or upregulated by *miR-1* overexpression or inhibition, respectively, however, unchanged by miRNA-masking the 3’UTR of the *Snap25* gene. SV exocytosis was inhibited by *miR-1* overexpression, which could be prevented by co-transfection with an anti-*miR-1* oligonucleotide fragment (AMO-1). The knockdown of *miR-1* by hippocampal stereotaxic injection of AMO-1 carried by a lentivirus vector (lenti-pre-AMO-1) led to the upregulation of SNAP-25 expression and prevented SV concentration in the synapses in the hippocampi of Tg mice. The application of GW4869 significantly reversed the increased *miR-1* level in the blood and hippocampi as well as reduced the SNAP-25 protein levels in the hippocampi of both Tg and LCA mice.

**Conclusion:**

The overexpression of *miR-1* in the heart attenuated SV exocytosis in the hippocampus by posttranscriptionally regulating SNAP-25 through the transportation of exosomes. This study contributes to the understanding of the relationship between cardiovascular disease and brain dysfunction.

## Background

The primary function of microRNAs (miRNAs) is posttranscriptionally regulating target protein expression by binding to their mRNA recognition sequences [1]. However, with the increasing number of miRNA studies, miRNAs were found to be stable in various body fluids (i.e., serum, plasma, saliva, urine, breast milk, and tears), and showed potential as biomarkers for various diseases [2–9]. More excitingly, miRNAs are also mediate trans-regulation between different cell types after these molecules are secreted into the interstitial space from donor cells and accepted by close recipient cells with functionally targeting capabilities through the actions of various transporters [10–13]; miRNAs even mediate long-distance metabolic regulation from adipose to the liver [14] and in cancer metastasis [15, 16]. However, whether miRNAs could mediate long-distance heart-brain communication and act as a link between heart disease and brain dysfunction is largely unknown, which would be very interesting.

**Fig. 1:**
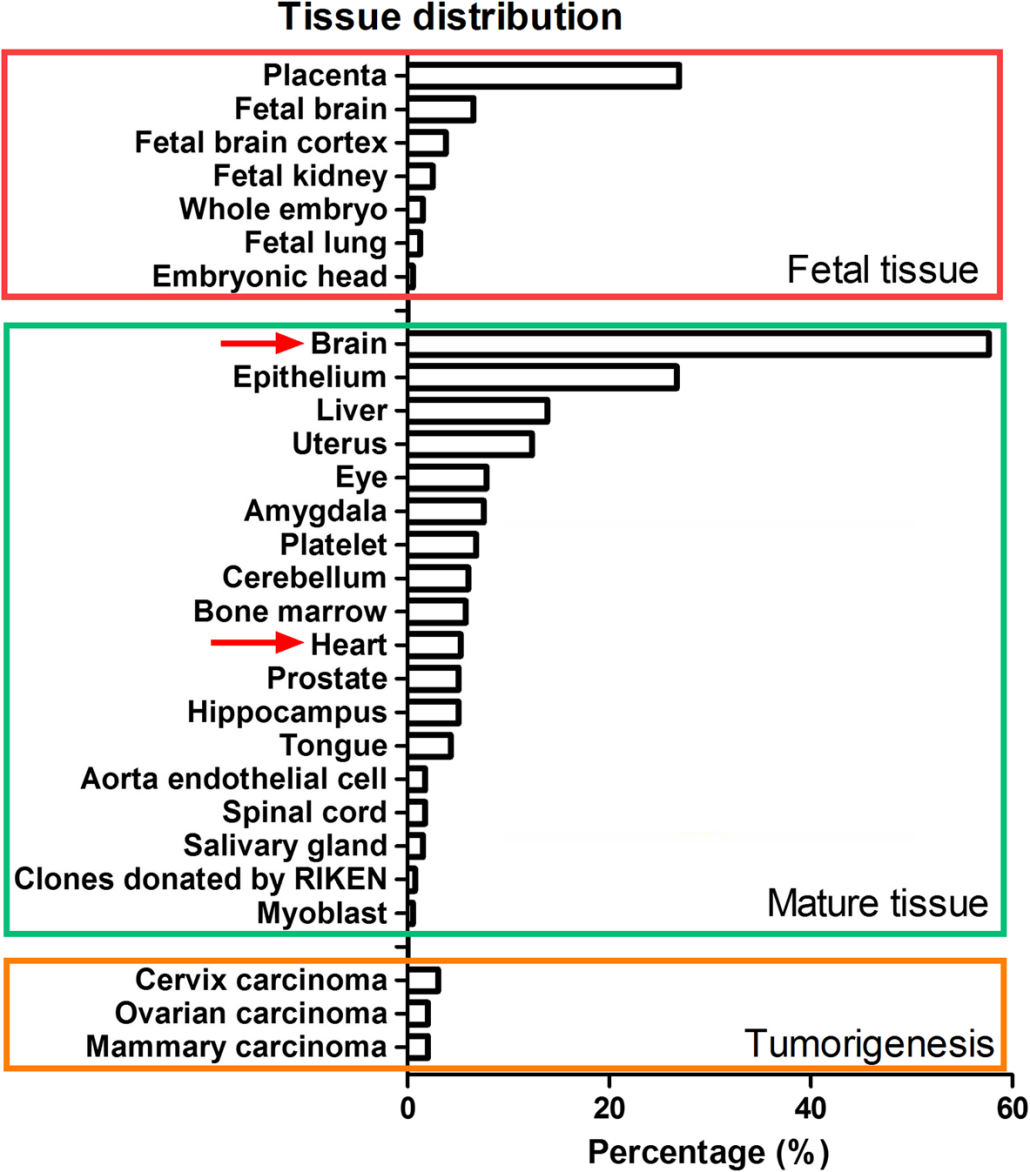
Distribution of *miR-1* targets in different tissues. By uploading the 398 target genes onto the David bioinformatics web site, we obtained the tissue distribution of 358 *miR-1* targets. All of the *miR-1* targets were classified into 3 parts: targets in foetal tissue, targets in mature tissue and targets associated with tumourigenesis. In each part, we analysed the percentage of *miR-1* targets in each kind of tissue. For example, in mature tissue, 57% of the targets are distributed in brain tissue and 5% are located in the heart (red arrow)

*MicroRNA-1* (*miR-1*) is a muscle-enriched miRNA that presents a hundred-fold lower level in the brain than in the heart [17, 18]. Interestingly, by bioinformatics analysis, we found that as a muscle-enriched microRNA, 57% of the targets of *miR-1* were distributed in the brain and only 5% of the targets were distributed in the heart of adult mice (Fig. 1). Our previous study demonstrated the behavioural abnormalities in a cardiac-specific *microRNA-1-2* (*miR-1-2*) overexpression transgenic (Tg) mouse model, which was associated with the downregulation of BDNF expression in the hippocampus [18]. Furthermore, a very recent study demonstrated that the overexpression of *miR-1* in the heart could induce neuronal microtubule damage [19]. These studies revealed the potential of cardiac-originated miRNAs to regulate brain function. Indeed, clarifying the underlying biological significance of heart-brain communication mediated by miRNAs would provide a new insight into association to the prevention of brain dysfunction.

Synaptic pathology is one of the prominent features of Alzheimer’s disease (AD) or vascular dementia (VaD) [20]. Considering the relationship between cardiovascular diseases and dementia [17, 21–25], we hypothesized that the overexpression of *miR-1* in the heart might be involved in heart disease-induced abnormal synaptic plasticity. The study will provide evidence to reveal a novel insight into the molecular mechanism of the heart-to-brain connection and disclose a potential communication between cardiac disease and brain disorder.

## Methods

### Animals

Adult male C57BL/6 mice and cardiac-specific *miR-1* overexpression transgenic (Tg) mice (6–7 months) were housed under a controlled temperature of 23 ± 1 °C and humidity of 55 ± 5%. The animals were maintained on a 12 h artificial dark-light cycle (lights on at 07:00 A.M.) with food (regular chow) and water ad libitum. All animal procedures were approved by the ethics committee of Harbin Medical University and carried out in accordance with the European Communities Council Directive of November 24, 1986 (86/609/EEC).

### *MiR-1* targets and bioinformatic analysis

We acquired mouse miRNA target genes from 7 miRNA target predicting tools, including PicTar, RNAhybrid, DIANA-microT, RNA22, miRBase Targets, miRanda and TargetScan. To improve the reliability of the predicted miRNA regulations, we only extracted the regulations that were predicted by at least two tools. We obtained 398 target genes. Finally, by uploading the 398 target genes onto the David bioinformatics web site, we obtained the tissue distribution of 358 *miR-1* targets. After double verification by literature citation, the percentages of the target gene locations were calculated.

### Generation of *miR-1* transgenic (Tg) mice

*MiR-1* Tg mice were generated as previously described [26]. Sexually immature female C57BL/6 mice (4–5 weeks of age) were used to obtain sufficient quantity of eggs (> 250) for microinjection. The Tg mice used in this study were 5th generation or later. Five- and six-month-old mice (5 M and 6 M) were used in this experiment.

### In situ hybridization (ISH)

First, 10 μm paraffin sections were prepared from the hippocampi and hearts of mice. After deparaffinization with xylene and ethanol, the sections were washed 3 times with a phosphate buffer solution (PBS) buffer for 5 min each. Then, proteinase K (40 μg/mL) was applied to deproteinate the sections for 20 min at room temperature. Second, after re-fixing in 4% paraformaldehyde(PFA) for 10 min and acetylating in 1.15% triethanolamine and 0.25% acetic anhydride, the sections were prehybridized in hybridization buffer (50% formamide, 5x standard saline citrate (SSC), 0.1% Tween-20, 50 μg/mL heparin, 500 μg/mL yeast tRNA, and 9.2 mM citric acid for adjustment to pH 6) for 2 h at 37 °C. The digoxigenin (DIG)-labelled detection probes mmu-*miR-1* (30 nM,: No. 21378–15) and scramble-*miR-1* (mis-*miR-1*, 30 nM, No.99004–15, Exiqon, Vedbaek, Denmark) were added to the slides and hybridized with the tissue overnight at 50 °C in a humidified chamber. The next day, the slides were washed twice with 5× SSC solution (20× SSC, 3 M NaCl, and 0.3 M sodium citrate) for 20 min each, and 3 times with 50% formamide at 37 °C for 20 min each and 5 times with TBS-T sequentially. After blocking for 1 h in blocking solution (2% goat serum, 2 mg/mL BSA, 0.1% Tween-20, and 1× PBS), the sections were incubated with anti-Digoxigenin-AP (1:1000, product No.11093274, Roche, Indianapolis, IN, USA) in blocking solution overnight at 4 °C. Finally, after washing 4 times with TBS containing 0.1% Tween-20, the slides were treated with developer buffer (0.1 M Tris, 0.1 M NaCl, 0.05 M MgCl_2_, 0.1%Tween, pH 9.5) for 20 min. Colour development was performed with 1-Step™ NBT/BCIP (product No.34042, Thermo Scientific, Rockford, USA). Images were captured using a microscope (AXIO, Zeiss, Germany).

### Mouse model of myocardial infarction

The method was described in a previous study [27]. The male C57BL/6 wild-type mice were divided into two groups: the sham group and the myocardial infarction (MI) group. Briefly, the mice were anaesthetized with sodium pentobarbital (40 mg/kg, intraperitoneal) and placed in the supine position with the upper limbs taped to the table. A 1-cm incision was made along the left side of the sternum, and the muscle layers of the chest wall were bluntly dissected to avoid bleeding. The thorax was cut open at the point of the most pronounced cardiac pulsation, the left anterior descending coronary artery was ligated with a 7-0 silk suture and then the chest was closed. Ischaemia was confirmed by an elevated ST segment of the electrocardiogram. Sham-operated control mice underwent the same surgical procedures except that the suture placed under the left coronary artery was not tied. All surgical procedures were performed under sterile conditions. After occlusion for 6 h, 1 d, 15 d and 30 d, hippocampi were collected for next experiments.

### Evaluation of ultrastructure morphology by transmission electron microscopy

For transmission electron microscopy detection, the mice were anaesthetized with sodium pentobarbital (100 mg/kg). The hippocampi were removed and immersed in stationary liquid (pH 7.3) containing 3% glutaraldehyde in 0.1 mmol/L sodium phosphate buffer and 0.45 mmol/L Ca^2+^. The tissue samples were then fixed in 2% osmic acid (OsO_4_) in PBS with 1.5% potassium ferricyanide. After dehydration with a concentration gradient of alcohol solutions, the tissues were embedded in Epon with propylene oxide as an intermediary solvent. Ultrathin sections were stained with uranyl acetate and lead citrate. The images were examined under a Hitachi H-7650 electron microscope (Hitachi, H-7650, Tokyo, Japan). To analysee vesicular distribution, 20 synapses without organelles near the presynaptic membrane were selected in each group (3 animals). The active zone was identified by a dark thickened presynaptic membrane with a visible synaptic cleft between the pre- and postsynaptic membranes. Both active zone length and the number of synaptic vesicles were measured using Image J software (National Institutes of Health, US). The total number of synaptic vesicles and vesicles located at 0 nm, 0–50 nm and 50–100 nm distances away from the active zone were counted in each selected pre-synapse [28].

### Oligonucleotide synthesis

*MiR-1* mimics and AMO-1 for rats were synthesized by Shanghai GenePharma Co., Ltd. (Shanghai, China). AMO-1 contains 2’-*O*-methyl modifications. In addition, scrambled RNA (mis-*miR-1*) was used as a negative control. *Snap25*-masking antisense oligodeoxynucleotides (ODNs) were synthesized by Shanghai Sangon Biotech Co., Ltd., China, and these ODNs complemented the position of 408–430 containing the binding sites of *miR-1* located at position 413–420 in the 3’ UTR of *Snap25*. The nucleotides or deoxynucleotides at both ends of the antisense molecules were locked by a methylene bridge connecting the 2’-*O* and 4’-C atoms. The sequences of these synthesized oligonucleotides are shown in Table 1.

**Table 1:** The sequences of the synthesized oligonucleotides

*miR-1* mimics	sense: 5’-UGGAAUGUAAAGAAGUGUGUAUGU-3′ antisense: 5’-AUACACACUUCUUUACAUUCCAAU-3’
AMO-1	5’-ACCUUACAUUUCUUCACACAUACA-3’
mis-*miR-1*	sense: 5’-UUCUCCGAACGUGUCACGUAA-3′ antisense: 5’-ACGUGACACGUUCGGAGAAUU-3’
*Snap25*-ODN	5’GUAGCUCUGUGGAAUGUCACAG-3’

### Construction of lentivirus vectors

To produce a *miR-1* antisense inhibitor, two single-stranded DNA oligonucleotides were designed as follows: (1) pre-AMO-1 (“top strand” oligo: tgctg ATACATACTTCTTTACATTCCAGTTTTGGCCACTGACTGACTGGAATGTAGAAGTATGTAT) and its complementary sequence (“bottom strand” oligo: cctgATACATACTTCTACATTCCAGTCAGTCAGTGGCCAAAACTGGAATGTAAAGAAGTATGTATc); (2) pre-mis-AMO-1 as a negative control (“top strand” oligo: tgctgAAATGTACTGCGCGTGGAGACGTTTTGGCCACTGACTGACGTCTCCACGCAGTACATTT) and its complementary sequence (“bottom strand” oligo: cctgAAATGTACTGCGTGGAGACGTCAGTCAGTGGCCAAAACGTCTCCACGCGCAGTACATTTc). Double-stranded oligonucleotides (ds oligos) were generated by annealing the above two strands with the pcDNA™6.2-GW/± EmGFP-miR vector and transforming the ligated construct into competent *Escherichia coli*, using a BLOCK-iT pol II miR RNAi expression vector and an EmGFP kit from Invitrogen (Shanghai, China). A pre-miRNA expression cassette was transferred to a Gateway® adapted destination vector utilizing Pol II promoters to form a new miRNA expression clone containing attR substrates. The vector was identified by analysing the plasmid sequence (Invitrogen, Shanghai, China). The lenti-pre-AMO-*miR-1* vectors used for the experiments (2.0 μL) contained 1.0 × 10^8^ transducing units (TUs)/mL. Virus suspensions were stored at − 80 °C until use and were briefly centrifuged and kept on ice immediately before injection.

### Stereotactic injection of lentiviral vectors

The mice were anaesthetized with sodium pentobarbital (40 mg/kg) by intraperitoneal injection and maintained under anaesthesia using 0.5–1.0% isoflurane. The depth of anaesthesia was monitored by detection of reflexes, heart rate and respiratory rate. After anaesthesia, the mice were placed on a stereotaxic frame (RWB Life Science Co. Ltd., China). Injections were administered in the CA1 area of the hippocampus using a 5-μL Hamilton syringe with a 33-gauge tip needle at − 2.2~ 2.5 mm below the surface of the dura. The injection coordinates relative to the bregma were as follows: anteroposterior (AP), − 2.8 mm; mediolateral (ML), ±3.0 mm; and dorsoventral (DV), 2.2–2.5 (Hamilton, Bonaduz, Switzerland). The needle was maintained in place for another 2 min after injection and was then withdrawn very slowly to avoid backflow of the solution. The accuracies of the injection sites were confirmed by stereotaxic injection of Evans blue (Sigma Chemical Co., St. Louis, Missouri, USA) directly into the hippocampus CA1 subfield [18, 29].

### Administration of GW4869 into MI and Tg mice

GW4869 (N,N’Bis[4-(4,5-dihydro-1H-imidazol-2-yl) phenyl]-3,3′-p-phenylene-bis-acrylamide dihydrochloride; MW:577.5 g/mol; Cayman Chemical) was first dissolved in the solution of DMSO as the storage solution at a concentration of 8 mg/mL, which was diluted to a concentration of 0.3 mg/mL by 0.9% NaCl before administration. The method of intraperitoneal injection of GW4869 was used in this study, and the single injection dosage was 200 μl at 0.3 mg/mL for each mouse. Equal volumes of 3.75% DMSO diluted with 0.9% NaCl were injected into mice as a control group. The first injection was performed on the day before LCA surgery for myocardial infarction mice and at the age of 6 M for Tg mice. Thereafter, GW4869 administration was performed 1 times per 2 d for 15 d, and the mice were sacrificed 24 h after the final injection for the further experiments.

### Blood sampling

Whole blood samples (0.5 mL per mouse) were collected from the hearts of the anaesthetized mice (sodium pentobarbital, 100 mg/kg, i.p.) via direct venous puncture. Blood was collected into a vacuum blood collection tube containing sodium citrate and was then carefully transferred into an RNase-free tube for RNA extraction [5, 17].

### Primary culture of neonatal rat hippocampal and cortical neurons (NRNs)

The primary culturing of NRNs was performed according to procedures that have been previously described in detail [30]. Briefly, hippocampal and cortical regions were collected from rat pups at postnatal day 0 (P0) and placed immediately into ice-cold PBS. The samples were then dissected and incubated in the presence of 0.125% trypsin (Gibco, USA) for 15 min in a water bath at 37 °C. After dispersion, cells were plated onto cell culture plates pre-coated with 10 μg/mL poly-D-lysine (Sigma, USA). The cells were maintained in culture media containing neurobasal medium (Gibco, USA) with 2% B27 supplement (Invitrogen, USA) and 10% foetal bovine serum (FBS, HyClone, Logan, UT) at a density of 1~ 3 × 10^5^ cells/cm^2^. The cultures were incubated in a 37 °C humidified chamber that was maintained at 5% CO_2_. The cells were fed by exchanging 50% of the culture media twice a week. The neurons were treated with 5 μM cytosine arabinoside (Sigma, USA) after 3 d in culture to inhibit astrocyte proliferation. For all experiments, neurons were used at 7–10 days after plating.

### Transfection procedures

A dose of 75 pmol/mL *miR-1*, *mis-miR-1*, AMO-1, *Snap25-ODN* siRNA or diethyl phosphorocyanidate (DEPC) water was transfected into NRNs using X-treme GENE siRNA transfection reagent (Cat.# 04476093001, Roche, USA) according to the manufacturer’s instructions [29]. The cells were collected for total RNA isolation or protein purification at 48 h post-transfection.

### Dual luciferase reporter assay

We cloned the full-length of 3’UTR of the *Snap25* gene to generate reporter vectors with miRNA binding sites. The full-length 3’UTR of *Snap25* was then amplified by PCR and cloned into the psi-CHECK™-2 luciferase expression vector containing Not1-Xho1 sites. HEK293T cells minimally expressing endogenous *miR-1* were used in the luciferase assay. These cells were cultured in DMEM with 10% FBS and 100 μg/mL penicillin/streptomycin. In vitro transfection and luciferase assays were conducted using HEK293T cells (plated at 40%~ 50% confluence and 20 μmol/L *miR-1*, AMO-1, or *mis-miR-1* as well as 0.5 μg psi-CHECK™-2-target DNA firefly luciferase vector, 1 μL blank plasmid and Lipofectamine 2000 (Invitrogen) transfection reagent according to the manufacturer’s instructions. After 48 h, the luciferase activity was measured with a dual luciferase reporter assay kit (Cat.#. E1910, Promega) and a luminometer (GloMax™ 20/20, Promega, USA). Nucleotide-substitution mutations in the 3’UTRs of *Snap25* were generated by direct oligomer synthesis. The sequences of constructs were verified [26, 29].

### TaqMan qRT-PCR

The levels of *miR-1* were quantified using a TaqMan® MicroRNA Reverse Transcription Kit (Cat.#4,366,596, Applied Biosystems) and TaqMan® Gene Expression Master Mix (the target sequences included UGGAAUGUAAAGAAGUGUGUAU (Cat.#002064) for rats and UGGAAUGUAAAGAAGUAUGUAU (Cat.#002222) for mice; Applied Biosystems), and U6 (Cat.# 001973, Applied Biosystems) was used as an internal control. The TaqMan qRT–PCR probes and primers for *miR-1* were designed by Invitrogen (USA). qRT-PCR was performed with a thermocycler (ABI Prism® 7500 fast, Applied Biosystems, Foster City, CA) for 40 cycles. The threshold cycle (Ct) was defined as the fractional cycle number at which the fluorescence passed the fixed threshold. PCR was performed as follows: (1) 95 °C for 10 min; (2) 95 °C for 15 s, followed by 60 °C for 1 min (repeat (2) for 40 cycles). The results were normalized against U6 expression levels using the δ–δ Ct method [26].

### Western blot analysis

Total protein samples for western blot analysis were extracted from cultured NRNs or the left ventricle and hippocampi of the mice after they were anaesthetized with sodium pentobarbital (100 mg/kg, i.p.). Mouse death was then confirmed by exsanguination according to a previously described method [31]. Hippocampi for primary cell culture were collected from neonatal Sprague-Dawley (SD) rats after the administration of 20% isoflurane and confirmation of death by cervical dislocation. Anti-SNAP-25 (1:1000, ab5666, Abcam, MA, USA), anti-VAMP-2 (1:10000, 104,211, Synaptic Systems, Gottingen, Germany) and anti-Syntaxin-1A (1:5000, 100,111, Synaptic Systems, Gottingen, Germany), anti-Munc-18 (1:1000, 116,002, Synaptic Systems, Gottingen, Germany), anti-CD63 (1:1000, ab193349, Abcam, MA, USA),were used as primary antibodies. β-actin (1:1000, G8795, Sigma, Saint Louis, MO, USA) was selected as an internal control. The blots were detected with an Odyssey Infrared Imaging System (LI-COR Biosciences, Lincoln, NE, USA). The final results were expressed as fold changes compared with the control values.

### Detection of synaptic vesicle exocytosis with FM1–43 dyes

FM1–43 fluorescence dye (T35356, Invitrogen, Oregon, USA) was dissolved in deionized, distilled water to make stock solutions (1 mg /mL) and stored at 2–8 °C. After the NRNs were cultured for 10–14 days in the cover glasses, the cell slides were placed in glass bottom of cell culture dishes (Nest, Cat. NO. 801002, China) and washed twice gently with 0.9% NaCl at 37 °C. First, the NRNs were incubated with an FM1–43 fluorescence working solution(10 μg/mL) dissolved in 0.9% NaCl for 3 min at room temperature to allow FM 1–43 binding to the outer membrane of NRNs. Then, 70 mM KCl dissolved in 0.9% NaCl was added to the FM1–43 loaded NRNs for 3 min at room temperature to internalize FM1–43 dye by endocytosis. Subsequently, the NRNs were washed again to remove the extracellular FM1–43 dye with 0.9% NaCl at 37 °C and then placed on a confocal microscope stage. The FM1–43 loaded NRNs were treated with 70 mM KCl solution to elicit synaptic vesicle exocytosis. The declined FM1–43 fluorescence dye signal due to the activated synaptic vesicle exocytosis was monitored by confocal microscopy (Olympus FV1000, Japan), and fluorescence images were acquired every 500 ms [8, 32].

### Statistical analysis

The data are described as the mean ± SEM. FM1–43 staining was performed using factorial ANOVA (split-plot design) after performing Mauchly’s test of sphericity with a *P* > 0.05. Post hoc analyses of significant main effects were further performed using Fisher’s least significant difference test (PLSD) for multiple comparisons. The independent sample test was calculated using the Levene test for equality of variance. If *P* > 0.05, then an independent Student’s t-test was used for the comparison between two groups. If *P* < 0.05, then the Kruskal-Wallis rank sum test was performed. One way ANOVA was performed for the comparison among multivariate groups and post hoc analyses of significant main effect ware further examined using Fisher’s PLSD for multiple comparisons. *P* < 0.05 was considered statistically significant. SAS 9.1 software (serial number: 989155; Institute Inc. China) was used for all statistical analyses.

## Results

### Abnormal synaptic vesicle distribution in the hippocampi of cardiac-specific *miR-1* overexpression Tg mice

**Fig. 2:**
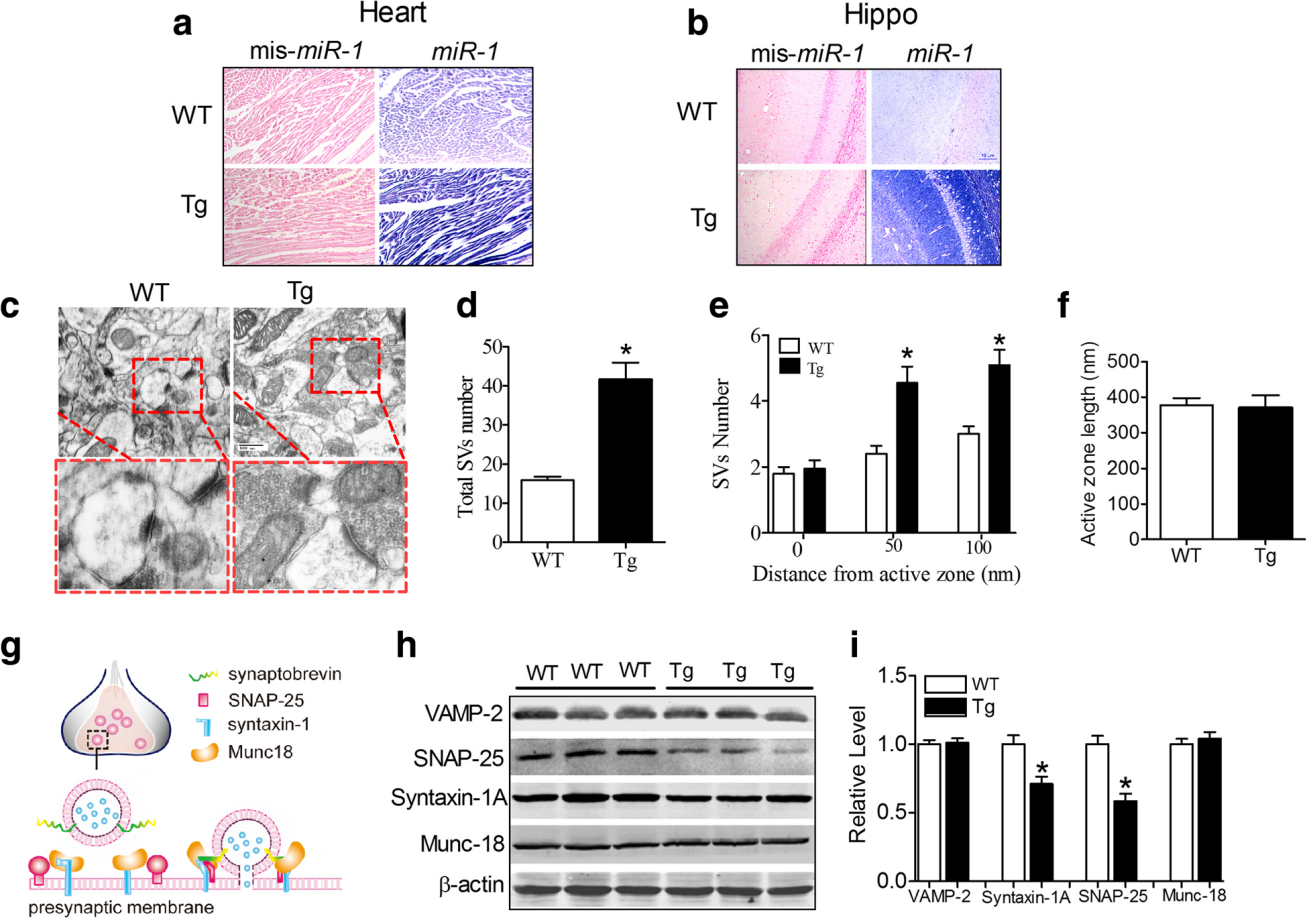
Abnormal synaptic vesicle distribution and related protein expression in the hippocampi of cardiac-specific *miR-1* overexpression Tg mice. **a-b** Elevation of *miR-1* levels in the hearts (**a**) and hippocampi (**b**) of Tg mice compared with WT mice by in situ hybridization (ISH) using DIG-lablled mmu-*miR-1* probes and scramble-*miR-1* (*mis-miR-1*) probes. The positive signal for mmu-*miR-1* probes was stained in blue. **c** Representative electron micrographs of Tg mice. **d** Morphometric analysis of the total number of SVs in the synapses of neurons from the hippocampi of Tg and WT mice. *P*_Levene_ < 0.0001, Kruskal-Wallis rank sum test: *P* < 0.0001. *n* = 20 synapses from 3 mice for each group. **e** Quantitative analysis of the distribution of SVs in WT and Tg nerve terminals. 0 nm shell: *P*_Levene_ = 0.828, Student’s t test: *t* = − 0.473, *P* = 0.639; 50 nm shell: *P*_Levene_ = 0.017, Kruskal-Wallis rank sum test: *P =* 0.001; 100 nm shell: *P*_Levene_ = 0.008, Kruskal-Wallis rank sum test: *P* < 0.0001. n = 20 synapses from 3 mice for each group. **f** Active zone length was not different between WT and Tg mice. *P*_Levene_ = 0.016, Kruskal-Wallis rank sum test: *P* = 0.844. n = 20 active zones from 3 mice for each group. **g** Schematic diagram of synaptic vesicle fusion. Under the stimulation of Ca^2+^, Munc-18 switches and binds to syntaxin-1 and assembles SNARE proteins into SNARE-complexes that promote SV fusion with presynaptic membranes. Synaptobrevin 2: VAMP-2. **h** Representative immunoblotting images of VAMP-2, SNAP-25, Syntaxin-1A, Munc-18. **i** Statistical analysis of VAMP-2: *P*_Levene_ = 0.478, Student’s t test: *t* = − 0.200, *P* = 0.845; SNAP-25: *P*_Levene_ = 0.562, Student’s t test: *t* = 3.463, *P* = 0.006; Syntaxin-1A: *P*_Levene_ = 0.670, Student’s t-test: *t* = 5.067, *P* < 0.0001. *n* = 6; Munc-18: *P*_Levene_ = 0.706, Student’s t-test: *t* = − 0.656, *P* = 0.527. *n* = 6. *P < 0.05; SV: synaptic vesicle

To observe whether the overexpression of *miR-1* in the heart could induce synaptic pathological remodelling in the brain, a mouse line for the cardiac-specific overexpression of *miR-1-2* driven by the α-myosin heavy chain (α-MHC) promoter was developed as described previously [26]. Similar to the previous study [18], *miR-1* expression was significantly increased in both the hearts (Fig. 2a) and hippocampi (Fig. 2b) of the Tg mice compared with that in age-matched wild type (WT) mice, as valuated by in situ hybridization technology.

Using transmission electron microscopy (TEM) examination, we found that the total number of synaptic vesicles (SVs) in the whole presynaptic area of the hippocampus was significantly increased (Fig. 2c and d). Interestingly, we found that SVs at 0 nm distances from the active zone were the same in the hippocampi of the mice in the two group but they were increased at 50 and 100 nm distances far from the active zone (Fig. 2c and e). However, no difference was observed in the active zone length between the groups (Fig. 2f). These results suggest a blunted fusion process between synaptic vesicles and presynaptic membranes in the hippocampi of the Tg mice. As displayed in Fig. 2g, SV exocytosis is a complex process that is dependent on the cooperation of multiple proteins including the SNARE complex, which is assembled by SNAP-25 (synaptosomal-associated protein 25), VAMP-2 (vesicle associated membrane protein 2, also known as synaptobrevin-2) and syntaxin-1 (also known as HPC-1), which determines the presynaptic release probability by controlling the fusion kinetics [7, 32]. We hence evaluated the expression of SNARE-complex proteins. We found that the SNAP-25 and syntaxin-1A protein levels were significantly reduced, while, VAMP-2 expression was unchanged (Fig. 2h & i). Furthermore, the protein level of Munc-18, which is considered to play a key role in presynaptic vesicle exocytosis by interacting with the SNARE complex [7], was unchanged in the hippocampi of the Tg mice compared with WT mice, (Fig. 2h & i),

### *MiR-1* targets the *Snap25* gene and attenuates synaptic vesicle exocytosis

**Fig. 3:**
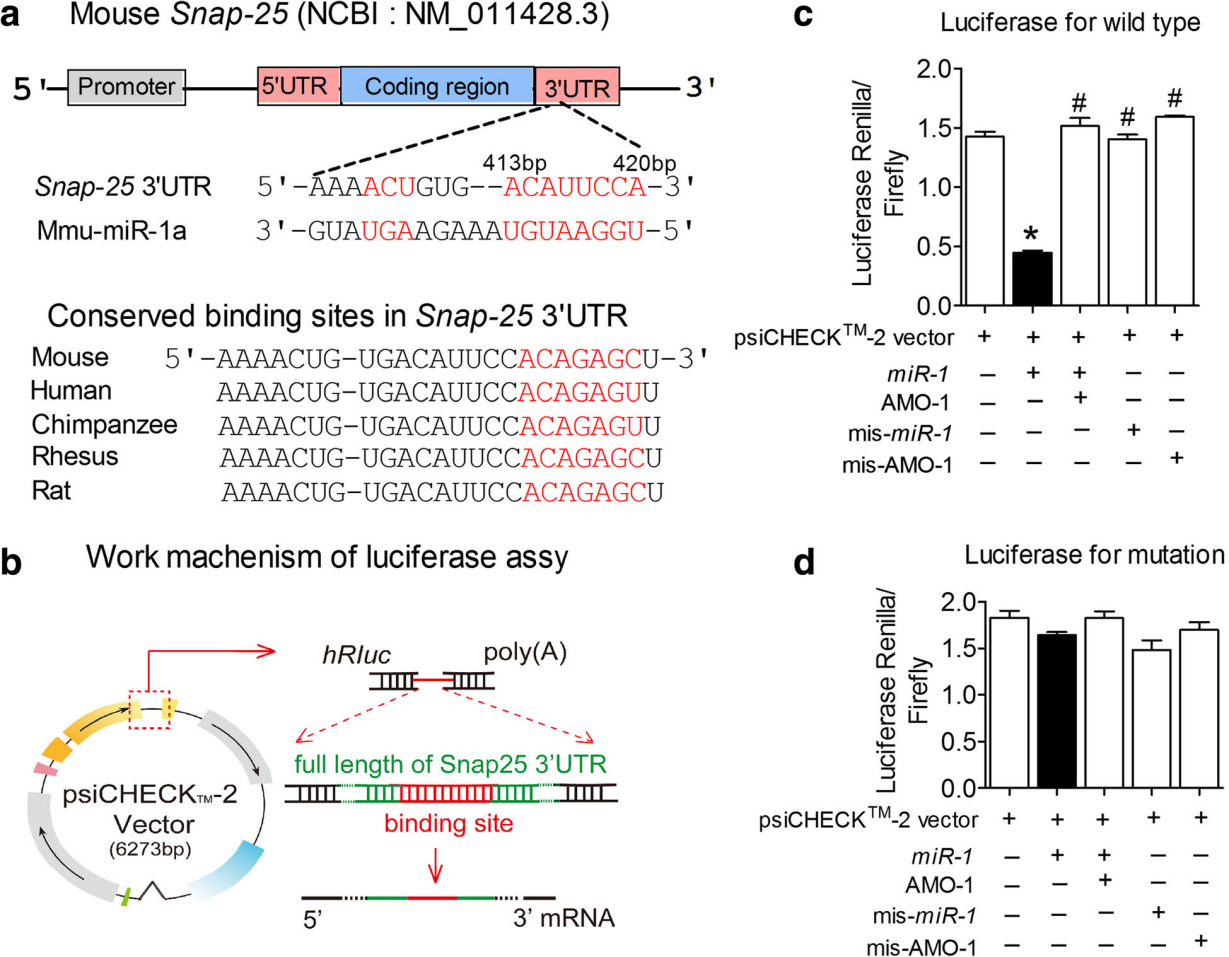
*Snap25* is a potential target of *miR-1.* **a** Complementarity between the *miR-1* seed sequence (5’end 2 ~ 8 nucleotides) and the 3’UTR of mouse’s *Snap25* gene as predicted by a computational and bioinformatics-based approach using the Targetscan 5.1 algorithm. Watson-Crick complementarity is marked by a red colour. **b** The mechanism of the luciferase assay. The full-length 3’UTR of *Sna*p25 was amplified by PCR and cloned into the psiCHECK™-2-control vector. **c-d** Luciferase reporter gene assay for interactions between *miR-1* and its binding site (**c**) and the mutated binding site (**d**) in the 3’UTR of the *Snap25* gene in HEK293T cells. HEK293T cells were transfected with psiCHECK™-2 vector, *miR-1* mimics, AMO-1, or negative control siRNAs (mis-*miR-1* and mis-AMO-1) using Lipofectamine 2000 (Wild type: *P*_Levene_ = 0.086, one-way ANOVA: F = 122.906, *P* < 0.0001; 3’UTR mut: *P*_Levene_ = 0.658, One-way ANOVA: F = 3.382, *P* = 0.054). *P < 0.05 versus psiCHECK™-2-control vector; ^#^*P* < 0.05, versus *miR-1*, *n* = 3 batches of cells for each group

We next aimed to clarify whether SNAP-25 and syntaxin-1A are the targets of *miR-1*. By performing computational analysis of miRNA databases (TargetScan 5.1 and miRanda), we found that there is a putative *miR-1* binding site in the 3’UTR of the *Snap25* gene at the site between 413 bp and 420 bp (Fig. 3a). However, we did not find the binding site of *miR-1* on the *Stx1a* gene (encoding Syntaxin-1A protein). To experimentally verify this finding, we first cloned the full-length 3’UTR of *Snap25* into a luciferase-expressing reporter vector (Fig. 3b) and then evaluated the effects of *miR-1* on reporter activity in HEK293T cells. The data showed that the co-transfection of the psiCHECK™-2 vector plasmid with *miR-1* consistently resulted in a reduction of luciferase activity compared with that observed in transfection of the plasmid alone (Fig. 3c, Renilla/firefly: 1.42 ± 0.08 in blank vs. 0.45 ± 0.03 in *miR-1*, *P* < 0.05). Application of a 2′-*O*-methyl antisense oligoribonucleotide to *miR-1* (AMO-1), a specific inhibitor of *miR-1*, eliminated the repressive effect of *miR-1* on the Renilla fluorescence signal (Fig. 3c, Renilla/firefly: 1.52 ± 0.116 in AMO-1 vs. 0.45 ± 0.03 in *miR-1*, *P* < 0.05). However, the negative control of both *miR-1* (mis-*miR-1*) and AMO-1 (mis-AMO-1) failed to influence *miR-1* action on the Renilla fluorescence signal (Fig. 3c). Furthermore, mutating the binding sites of the *Snap25* gene abolished the effect of *miR-1* (Fig. 3d), suggesting that these binding sites contribute to the repressive effects of *miR-1*.

**Fig. 4:**
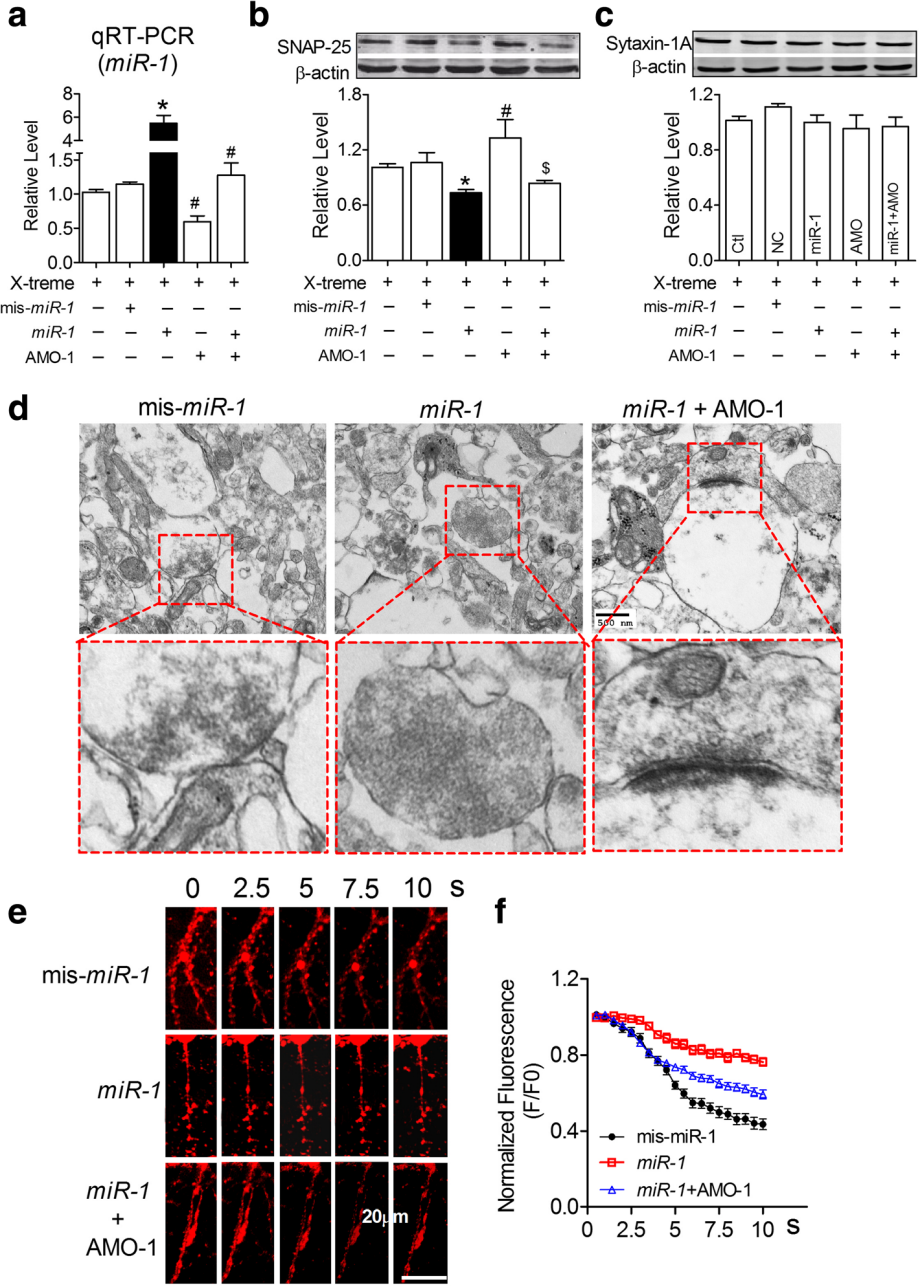
*MiR-1* inhibited SNAP-25 protein expression and presynaptic vesicle release. **a** Verification of *miR-1* in NRNs after transfection. *P*_Levene_ = 0.001, One-way ANOVA: F = 41.566, *P* < 0.0001; Fisher’s PLSD test: *P*_*mis-miR-1*: *miR-1*_ < 0.0001, *P*_*miR-1*: AMO-1_ < 0.0001. *n* = 3 batches of cells for each group. **b** Expression of SNAP-25 protein in NRNs was downregulated by *miR-1* determined by western blot analysis. *P*_Levene_ = 0.32, One-way ANOVA: F = 4.667, *P* = 0.008; Fisher’s PLSD test: *P*_*mis-miR-1*: *miR-1*_ = 0.04, *P*_*miR-1*: AMO-1_ = 0.001. *n* = 5 batches of cells for each group. **c** The expression of syntaxin-1A protein in NRNs was not influenced by *miR-1*. *P*_Levene_ = 0.151, One-way ANOVA: F = 1.033, *P* = 0.422. **d** Morphologic change of the transfected primary cultured neurons. **e** Representative FM1–43 fluorescent signalling changes in each group after 70 mM KCl stimulation. **f** Co-transfection of AMO-1 with *miR-1* improved the decline in FM1–43 signalling decline (F/F0) in NRNs compared with transfection of *miR-1* alone. *χ*^*2*^_Mauchly_ = 1652.465, *P* < 0.0001; F_total (19, 1407)_ = 48.263, *P* < 0.0001; Fisher’s PLSD test: *P*_*mis-miR-1: miR-1*_ < 0.0001, *P*_*miR-1: miR-1* + AMO-1_ < 0.0001, *P*_*mis-miR-1*: *miR-1* + AMO-1_ < 0.0001. *n* = 25 neurons from 3 batches of cells for each group

To observe whether *miR-1* regulates the expression of SNAP-25, we transfected *miR-1* and/or AMO-1 directly into NRNs using the X-treme GENE siRNA transfection reagent. The successful transfection of *miR-1* and/or AMO-1 was confirmed by *miR-1* levels in NRNs detected by qRT-PCR (Fig. 4a). Based on this condition, by immunoblotting analysis, we found that *miR-1* effectively inhibited SNAP-25 protein expression by nearly 30% relative to the level of the control group, whereas the scrambled negative control mis-*miR-1* failed to affect the protein level (Fig. 4b). In contrast, AMO-1 rescued the *miR-1*-mediated downregulation of SNAP-25 (Fig. 4b). As predicted, the overexpression of *miR-1* did not regulate syntaxin-1A expression in NRNs after transfection with *miR-1* and/or AMO-1 (Fig. 4c). To observe whether *miR-1* could influence SV number and distribution in vitro, TEM detection was performed. We observed a significantly increased number of SVs in the active zone of NRNs after the transfection of *miR-1* for 48 h, however, co-transfection of AMO-1 with *miR-1* blocked the *miR-1-*mediated aggregation of synaptic vesicles inside the synapses (Fig. 4d). FM1–43 fluorescence dyes are widely used to image vesicle exocytosis and endocytosis [8]. We observed that the FM1–43 fluorescent signal in NRNs transfected with mis-*miR-1* gradually decreased after 70 mM KCl stimulation (Fig. 4e). The ratio of F/F0 was reduced to 0.5 at 5 s, and further decreased to 0.4 at 10 s after KCl stimulation (Fig. 4f). The transfection of *miR-1* markedly blocked the attenuated FM1–43 fluorescent signal induced by KCl, which was reversed by the co-transfection of AMO-1 (Fig. 4e & f).

**Fig. 5:**
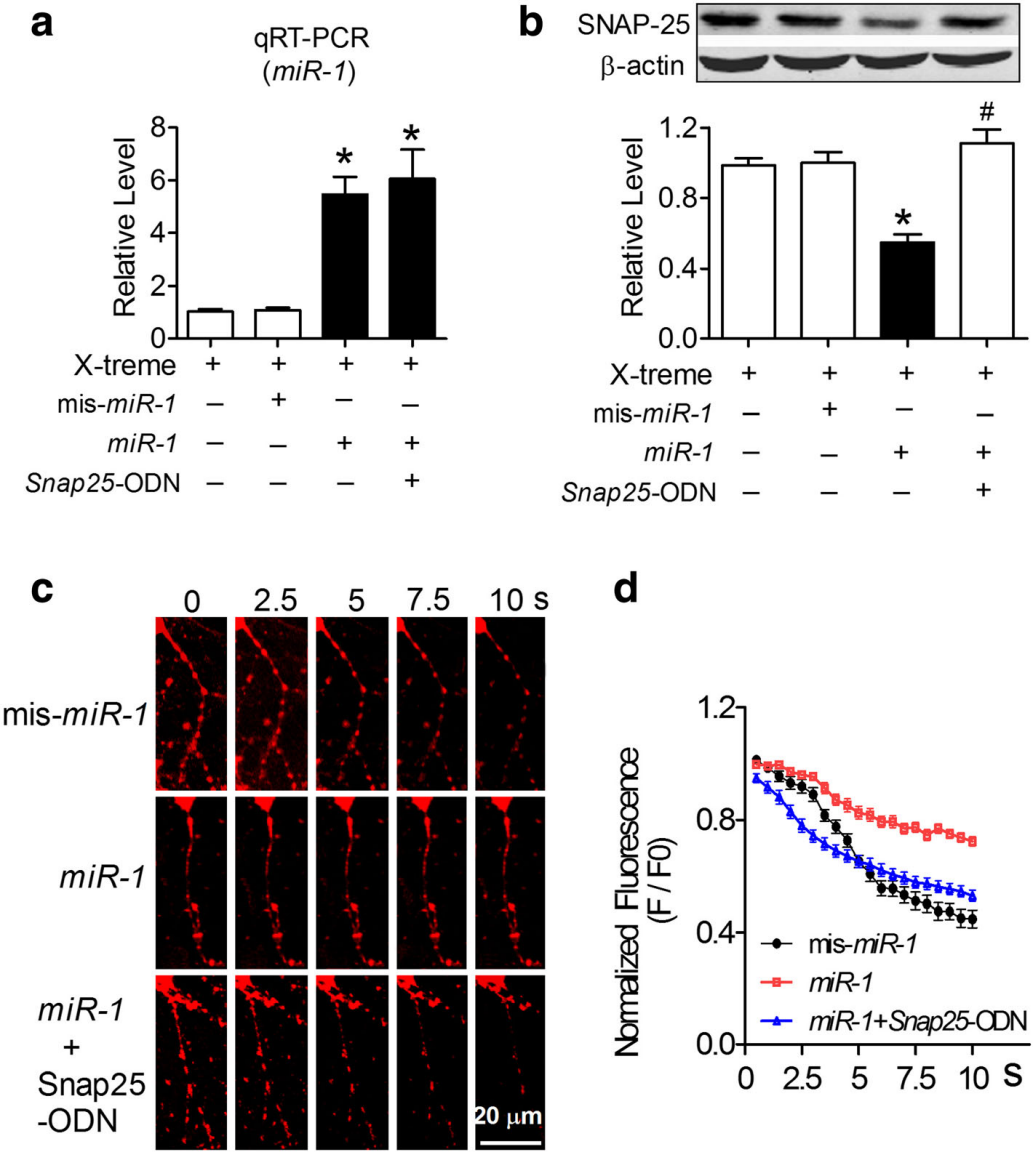
*MiR-1* influences vesicle exocytosis by inhibiting the expression of SNAP-25 directly. **a** *MiR-1* levels in NRNs transfected with *miR-1* and *Snap25*-ODN. *P*_Levene_ = 0.041, one-way ANOVA: F = 17.566, *P* = 0.001; Fisher’s PLSD test: *P*_*mis-miR-1*: *miR-1*_ = 0.01, *P*_*mis-miR-1*: *Snap25-ODN*_ = 0.001. *n* = 3 batches of cells for each group. **b** Derepression of SNAP-25 by *Snap25*-ODN in NRNs co-transfected with *miR-1*. *P*_Levene_ = 0.515, one-way ANOVA: F = 18.101, *P* = 0.001; Fisher’s PLSD test: *P*_*mis-miR-1*: *miR-1*_ = 0.01, *P*_mis-*miR-1*: *Snap25-ODN*_ < 0.0001. *P < 0.05 versus *mis-miR-1*-control vector; ^#^*P* < 0.05, versus *miR-1. n* = 3 batches of cells for each group. **c** The FM1–43 dye fluorescence change of NRNs co-transfected with *miR-1* and *Snap25-ODNs*. **d** *Snap25*-ODN failed to improve the decline in FM1–43 signalling (F/F0) in NRNs transfected with *miR-1* alone. *χ*^*2*^_Mauchly_ = 1935.529, *P* < 0.0001; F_total (19, 1407)_ = 35.946, *P* < 0.0001; Fisher’s PLSD test: *P*_*mis-miR-1*: *miR-1*_ < 0.0001, *P*_*miR-1: miR-1* + *Snap25-ODN*_ < 0.0001, *P*_*mis-miR-1*: *miR-1* + *Snap25-ODN*_ = 0.730. *n* = 25 neurons from 3 batches of cells for each group

To clarify whether the observed changes in SNAP-25 expression and exocytosis in cultured NRNs are the direct results of *miR-1* action on the binding sites of this genes, an miRNA-masking antisense ODN fragment (miR-mask) was designed from the 3’UTR of the *Snap25* gene sequence. The miR-mask was named *Snap25-ODN*, which acts on a specific binding site between 408 ~ 430 bp of this gene and minimally influences the effects of *miR-1* on other target genes and other binding sites of this gene [33]. As expected from their principle action, *Snap25-ODN*, unlike AMO-1, did not affect the *miR-1* level in cells co-transfected with *miR-1* (Fig. 5a). As predicted, *Snap25-ODN* inhibited the action of *miR-1* on SNAP-25 expression (Fig. 5b), suggesting that *miR-1* post-transcriptionally regulated SNAP-25 expression by binding to a site located at 413 ~ 420 bp in the 3’UTR of the *Snap25* gene. Furthermore, *Snap25-ODN* successfully prevented the *miR-1* overexpression-induced impairment of vesicle exocytosis (Fig. 5c & d). All these results suggested that *miR-1* overexpression triggered impaired synaptic vesicle exocytosis, mainly by reducing the SNAP-25 protein level.

**Fig. 6:**
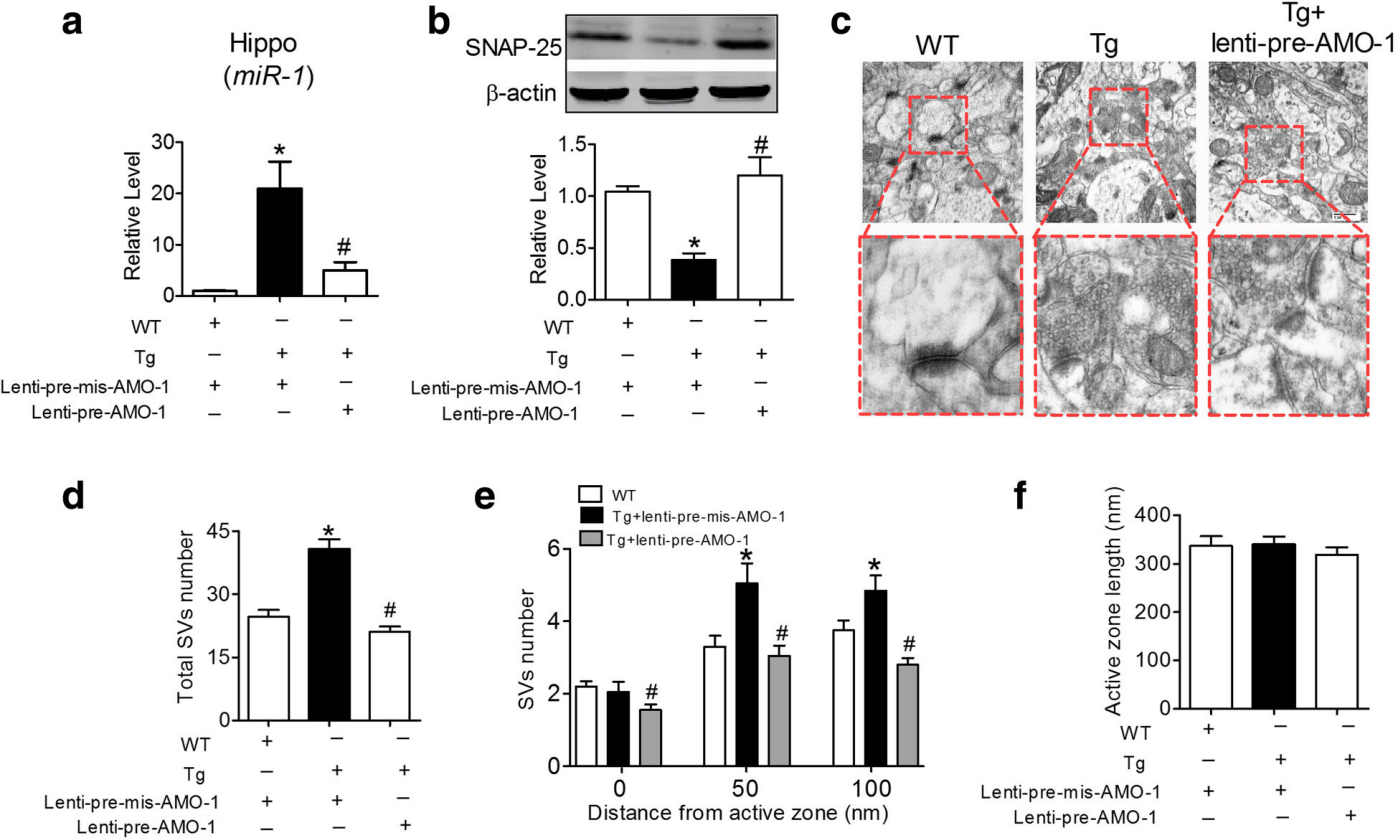
Stereotaxic injection of lenti-pre*-*AMO-1 into the hippocampi of Tg mice improved SV distribution. **a** Stereotaxic injection of lenti-pre*-*AMO-1 into the CA1 domain reduced the *miR-1* level in the hippocampi of Tg mice as determined by qRT-PCR analysis. *P*_Levene_ = 0.01, one-way ANOVA: F = 10.872, *P* = 0.01; Fisher’s PLSD test: *P*_WT:Tg_ = 0.005, *P*_Tg + lenti-pre-mis-AMO-1::Tg + lenti-pre-AMO-1_ = 0.012. *n* = 3 batches of cells for each group. **b** Stereotaxic injection of lenti-pre*-*AMO-1 into the hippocampi of Tg mice derepressed the expression of SNAP-25 protein. *P*_Levene_ = 0.004, one-way ANOVA: F = 15.700, *P* < 0.001; Fisher’s PLSD test: *P*_WT:Tg_ = 0.001, *P*_Tg + lenti-pre-mis-AMO-1:Tg + lenti-pre-AMO-1_ < 0.0001. *n* = 6 animals for each group. **c** Representative electron micrographs showing the effect of stereotaxic injection of lenti-pre*-*AMO-1 on SVs distribution at presynaptic active zone of Tg mice. **d** Lenti-pre*-*AMO-1 prevented an increase in the total number of SVs in the hippocampal synapses of Tg mice. *P*_Levene_ < 0.003, one-way ANOVA: F = 33.161, *P* < 0.0001; Fisher’s PLSD test: *P*_WT:Tg_ < 0.0001, *P*_Tg + lenti-pre-mis-AMO-1:Tg + lenti-pre-AMO-1_ < 0.0001. *n* = 20 active zones from 3 mice for each group. **e** Lenti-pre*-*AMO-1 redistributed SV location in Tg mice nerve terminals. 0 nm shell: *P*_Levene_ < 0.012, one-way ANOVA: F = 2.928, *P* = 0.062; Fisher’s PLSD test: *P*_WT:Tg_ = 0.596, *P*_Tg + lenti-pre-mis-AMO-1: Tg + lenti-pre-AMO-1_ = 0.024; 50 nm shell: *P*_Levene_ = 0.044, one-way ANOVA: F = 7.517, *P* = 0.01; Fisher’s PLSD test: *P*_WT:Tg_ = 0.003, *P*_Tg + lenti-pre-mis-AMO-1:Tg + lenti-pre-AMO-1_ = 0.001; 100 nm shell: *P*_Levene_ = 0.055, one-way ANOVA: F = 11.161, *P* < 0.0001; Fisher’s PLSD test: *P*_WT:Tg_ = 0.014, *P*_Tg + lenti-pre-mis-AMO-1:Tg + lenti-pre-AMO-1_ < 0.0001. n = 20 synapses s from 3 mice for each group. **f** Lenti-pre*-*AMO-1 did not affect the length of active zone. *P*_Levene_ = 0.551, one-way ANOVA: F = 0.404, *P* = 0.670; Fisher’s PLSD test: *P*_WT:Tg_ = 0.940, *P*_Tg + lenti-pre-mis-AMO-1:Tg + lenti-pre-AMO-1_ = 0.419. n = 20 active zones from 3 mice for each group. *P < 0.05 versus WT; ^#^*P* < 0.05 versus Tg

Furthermore, to determine whether *miR-1* is involved in the declined synaptic vesicle exocytosis in the Tg mice, anti-*miR-1* oligonucleotide fragments carried by a lentivirus vector (lenti-pre-AMO-1) were stereotaxically injected directly into the CA1 area of the hippocampus bilaterally in the Tg mice at 5 months. The *miR-1* levels in the hippocampi of the Tg mice were successfully inhibited by lenti-pre-AMO-1 after injection for 2 months (Fig. 6a). Similar to observations in the in vitro experiments (Fig. 4b), a reduction in SNAP-25 protein levels was observed in the hippocampi of the Tg mice with lenti-pre-mis-AMO-1 which was effectively prevented by lenti-pre-AMO-1 injection (Fig. 6b)*.* Furthermore, lenti-pre-AMO-1 significantly reversed the increased SV number in the Tg mice (Fig. 6c & d) and attenuated SV redistribution at the presynaptic active zone in the hippocampi of the Tg mice (Fig. 6c & e). In this way, lenti-pre-AMO-1 injection did not affect the active zone length compared with that in the uninjected Tg mice (Fig. 2f). These data indicated that the hippocampal overexpression of *miR-1* impaired SV exocytosis in Tg mice was associated with the decreased expression of SNAP-25 protein in the hippocampi.

### The cardiac *miR-1* overexpression-mediated attenuation of SV exocytosis involves exosomes

**Fig. 7:**
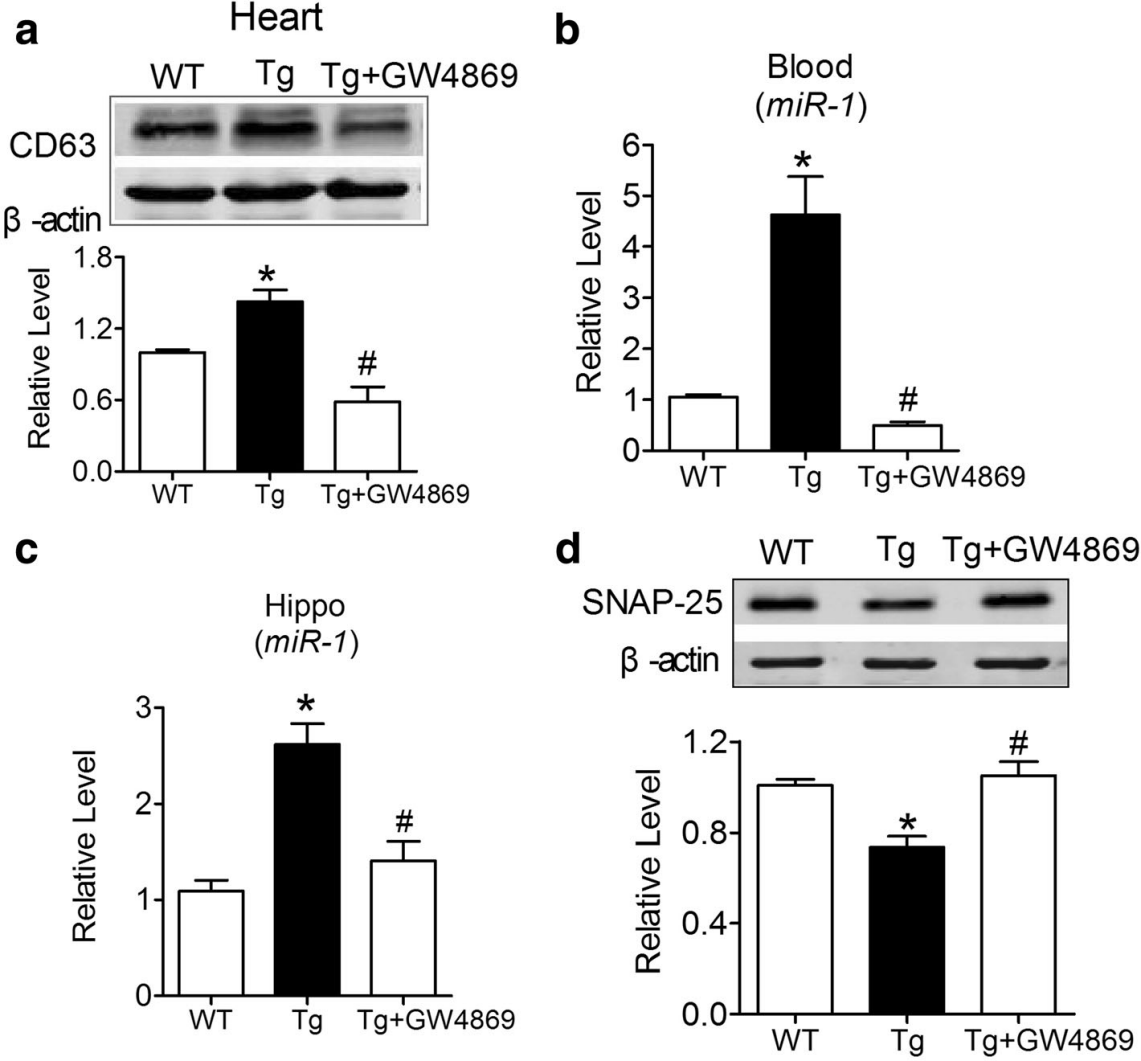
Inhibition of exosome generation derepressed the expression of SNAP-25 protein in the hippocampi of Tg mice. **a** Intraperitoneal injection of GW4869 inhibited CD63 expression in the heart of Tg mice at the age of 6 M compared with age-matched Tg mice, as measured by western blotting. *P*_Levene_ = 0.419, one-way ANOVA: F = 12.778, *P* = 0.001; Fisher’s PLSD test: *P*_WT:Tg_ = 0.002, *P*_Tg::GW4869_ = 0.001. *n* = 5 animals for each group. **b-c** Intraperitoneal injection of GW4869 prevented the elevation of *miR-1* level in the blood (**b**: P_Levene_ < 0.001, one-way ANOVA: F = 26.140, *P* < 0.0001; Fisher’s PLSD test: *P*_WT:Tg_ < 0.0002, *P*_Tg::GW4869_ < 0.0001. *n* = 6 animals for each group) and hippocampi (**c**: P_Levene_ < 0.001, one-way ANOVA: F = 7.756, *P* = 0.011; Fisher’s PLSD test: *P*_WT:Tg_ = 0.004, *P*_Tg::GW4869_ = 0.0181. *n* = 4 animals for each group) of Tg mice, as measured by qRT-PCR. **d** Intraperitoneal injection of GW4869 derepressed SNAP-25 expression in the hippocampi of Tg mice, as measured by western blotting. *P*_Levene_ = 0.419, one-way ANOVA: F = 12.778, *P* = 0.001; Fisher’s PLSD test: *P*_WT:Tg_ = 0.002, *P*_Tg::GW4869_ = 0.001. *n* = 6 animals for each group. *P < 0.05 versus WT; ^#^*P* < 0.05 versus Tg

We next examined why the overexpression of *miR-1* in the heart affects SV exocytosis in the brain. Studies have reported that miRNAs could be secreted into the extracellular space of donor cells and then transported and accepted by recipient cells with functional targeting capabilities [3, 11]. As one kind of miRNA transporter, exosomes not only contain up to 121 miRNAs, including *miR-1*, *miR-15*, *miR-16*, *miR-17*, *miR-18*, *miR-181*, *miR-375*, *lin-4* and *let-7* [14], but also show an increase in the serum of the infarcted heart of rats [19, 34]. We hence speculated that exosomes might participate in this process as transporters. To elucidate this issue, we applied GW4869 by intraperitoneal injection to inhibit sphingomyelinase, which was reported to inhibit exosome generation [17]. As illustrated in Fig. 7a, we observed that GW4869 significantly inhibited the reduction in CD63, a marker of exosomes, in Tg mouse hearts, suggesting that the generation of exosomes in the heart was prevented. As predicted, the *miR-1* level in the blood (Fig. 7b) and hippocampi (Fig. 7c) of Tg mice were both decreased in Tg mice after GW4869 intraperitoneal injection. Notably, the injection of GW4869 significantly reversed the reduction in SNAP-25 protein in the hippocampi of Tg mice (Fig. 7d).

**Fig. 8:**
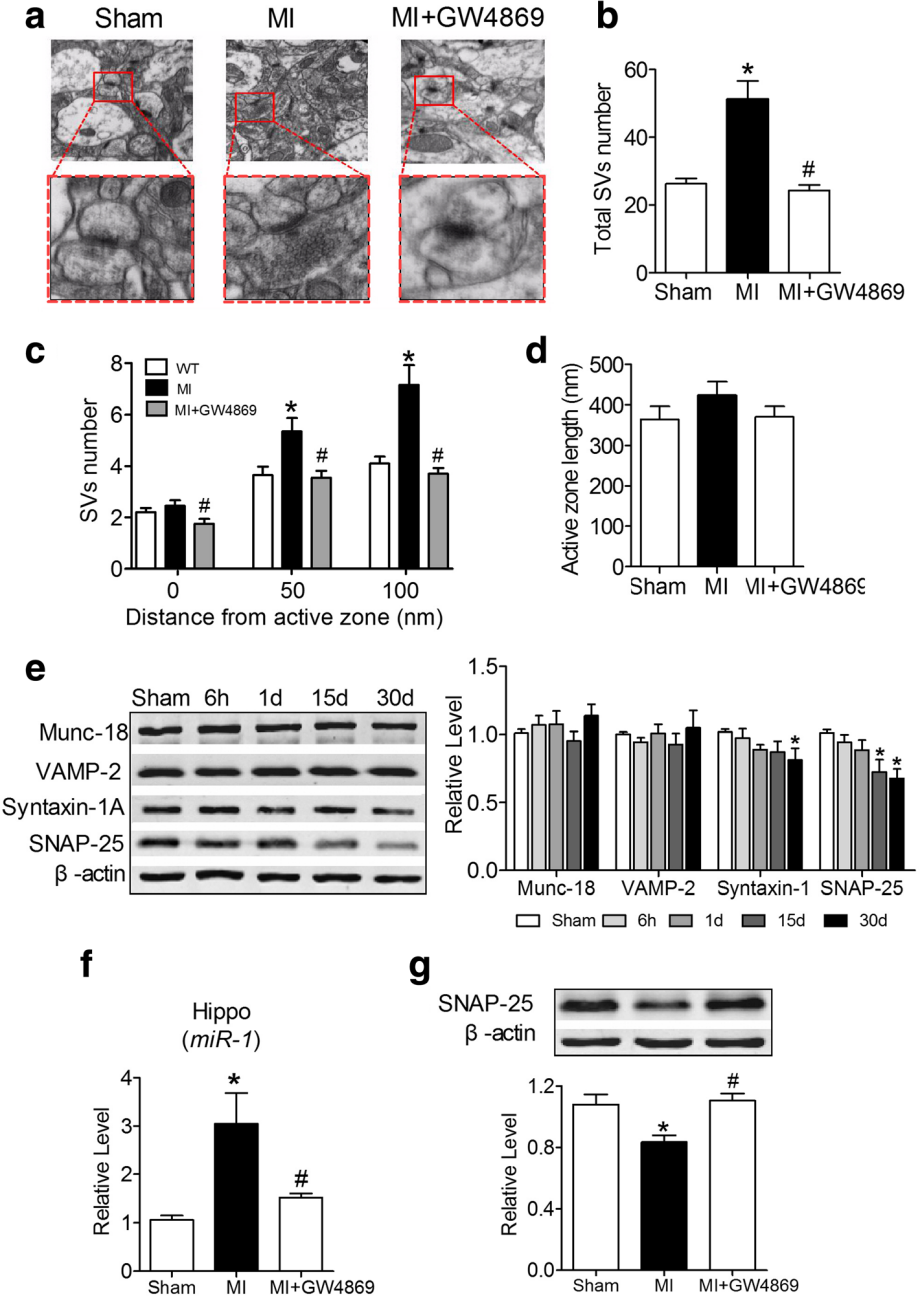
Inhibition of exosome generation improves MI disrupted SV distribution. **a** Intraperitoneal injection of GW4869 prevented the synaptic vesicles accumulation at the presynaptic active zone in hippocampal neurons of MI mice. **b** GW4869 prevented an increase of the total SV number in the synapses in the hippocampi of MI mice. P_Levene_ < 0.0001, one-way ANOVA: F = 19.799, *P* < 0.0001; Fisher’s PLSD test: *P*_sham: MI_ < 0.0001, *P*_MI:GW4869_ < 0.0001. n = 20 synapses from 3 mice for each group. **c** GW4869 redistributed SVs at the active zones of synapses in the nerve terminals of Tg mice. 0 nm shell: *P*_Levene_ = 0.669, one-way ANOVA: F = 3.424, *P* = 0.039; Fisher’s PLSD test: *P*_sham: MI_ = 0.360, *P*_MI:GW4869_ = 0.012; 50 nm shell: *P*_Levene_ = 0.016, one-way ANOVA: F = 6.703, *P* = 0.002; Fisher’s PLSD test: *P*_sham: MI_ = 0.003, *P*_MI:GW4869_ = 0.002; 100 nm shell: *P*_Levene_ < 0.0001, One way ANOVA: F = 14.785, *P* < 0.0001; Fisher’s PLSD test: *P*_sham: MI_ = 0.0001, *P*_MI:GW4869_ < 0.0001. n = 20 synapses from 3 mice for each group. **d** GW4869 did not affect the length of active zone in hippocampi of MI mice. *P*_Levene_ = 0.271, one-way ANOVA: F = 1.084, *P* = 0.345; Fisher’s PLSD test: *P*_sham: MI_ = 0.185, *P*_MI:GW4869_ = 0.885. n = 20 active zones from 3 mice for each group. **e** Expression of SV related proteins in the hippocampi of MI mice. Left: sample bands of immunoblotting images. Right: statistical analysis of Munc 18, VAMP-2, syntaxin- 1A and SNAP-25. *P < 0.05 (**f**) Intraperitoneal injection of GW4869 prevented the elevation of *miR-1* level in the hippocampi of MI mice, as measured by qRT-PCR. *P*_Levene_ < 0.0001, one-way ANOVA: F = 7.756, *P* = 0.011; Fisher’s PLSD test: *P*_sham: MI_ = 0.004, *P*_MI:GW4869_ = 0.018. *n* = 4 animals for each group. **g** Intraperitoneal injection of GW4869 prevented the decreased SNAP-25 expression in the hippocampi of MI mice, as measured by western blotting. *P*_Levene_ = 0.265, one-way ANOVA: F = 7.647, *P* = 0.011; Fisher’s PLSD test: *P*_sham: MI_ = 0.010, *P*_MI:GW4869_ = 0.006. *n* = 4 animals for each group. *P < 0.05 versus WT; ^#^*P* < 0.05 versus Tg

Importantly, *miR-1* has been reported to be increased in the blood of both acute myocardial infarction (AMI) rats and patients [5, 35, 36] and hippocampi of myocardial infarction (MI) mice [19]. Could MI also induce impaired presynaptic plasticity and share the same mechanism as that in Tg mice? We first developed an MI animal model by ligating of the left coronary artery (LCA) for 30 days. Similar to the observation in experiments of Tg mice (Figs. 2c & 6c), the number of SVs was significantly increased in MI mice compared with that in sham mice (Fig. 8a & b), and SVs were more concentrated at sites of 50 nm and 100 nm shells from the active zone in MI mice compared with those in age-matched sham mice (Fig. 8a and c). Strikingly, all these changes were prevented by intraperitoneal injection of GW4869 (Fig. 8a-c). However, neither MI nor GW4869 affected the active zone length (Fig. 8d). We then assessed the expression of synaptic fusion-related protein in the hippocampi of MI mice. We found that, consistent with Tg mice, both Munc-18 and VAMP-2 protein levels were unchanged compared with those in the sham control (Fig. 8e). However, SNAP-25 protein expression was significantly reduced from the 15th to the 30th day after LCA (Fig. 8e). Interestingly, although the syntaxin-1A protein level was decreased in the hippocampi of MI mice on the 30th day of LCA, the level was not changed before 15 d (Fig. 8e). Importantly, the intraperitoneal injection of GW4869 into MI mice markedly prevented the increased *miR-1* levels (Fig. 8f) and decreased SNAP-25 expression (Fig. 8g) in the hippocampi of MI mice. All these data suggested that the cardiac *miR-1* overexpression-mediated attenuation of synaptic vesicle exocytosis involves exosomes.

## Discussion

**Fig. 9:**
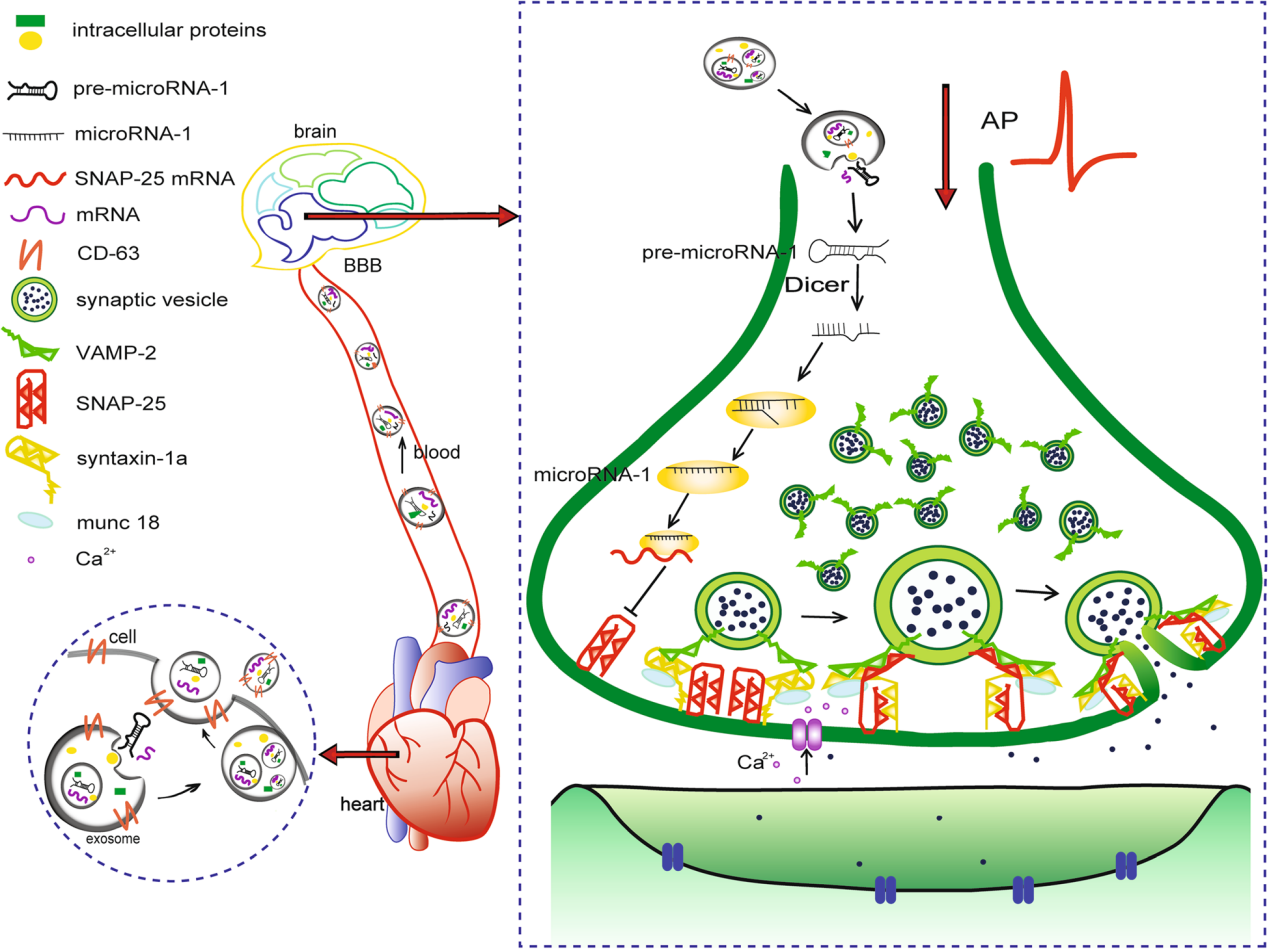
Schematic diagrams for the mechanism of the cardiac-specific *miR-1* overexpression-induced decline in synaptic vesicle exocytosis. Overexpressed *miR-1* in the hearts of Tg and MI mice were secreted into the extracellular space carried by exosomes. Exosomes entered the blood and were transported into the brain through blood circulation. After exosomes enter the brain, they were accepted by neurons and inhibited the expression of SNAP-25 by binding with the target in the 3’UTR of the *Snap*25 gene. The inhibition of SNAP-25 results in the impairment of vesicle exocytosis through impeding SNARE-complex formation

In the present study, we reported that *miR-1* overexpression in the heart could result in the attenuation of SV exocytosis, which is controlled by increased *miR-1* levels in the hippocampus originating from exosome-mediated transportation from the heart to the brain through circulation. The molecular mechanism involves *miR-1* targeting the 3’UTR of the *Snap25* gene and post-transcriptionally inhibiting the expression of SNAP-25 protein (Fig. 9). This study improved our understanding of how heart disease induces brain dysfunction at the miRNA level.

The link between cardiac diseases and cognitive deterioration has been accepted from the concept of “cardiogenic dementia” which was proposed in the late 1970s [37]. However, most studies have focused on cardiac disease-induced chronic brain hypoperfusion (CBH) due to the reduction of cardiac output [22, 24, 38]. Whether heart abnormalities alone could lead to brain damage and even affect cognition is elusive. Using the cardiac-specific overexpression of *miR-1-2* in transgenic mice driven by α-MHC, our previous study demonstrated that the overexpression of *miR-1* in the hearts of Tg and MI mice could increase its expression in the hippocampus and impaire cognition by post-transcriptionally inhibiting BDNF expression [18, 39]. Because synaptic pathology is a prominent feature of Alzheimer’s disease (AD) or vascular dementia (VaD) [20], we propose that the cardiac overexpression of *miR-1* affects synaptic plasticity. Using TEM technique, we observed an increase in *miR-1* levels and SV redistribution in the hippocampi of Tg mice, and this phenomenon was reversed by the stereotaxic injection of lenti-pre-AMO-1. This result suggests that the cardiac-specific overexpression of *miR-1* could impair synaptic plasticity, which may associate with increased *miR-1* levels in the hippocampus. To verify this hypothesis, we performed in vitro experiments and demonstrated that the overexpression of *miR-1* led to more synaptic vesicle retention at presynaptic synaptosomes and attenuated KCl-induced FM1–43 fluorescent signal decline, which were prevented by the *miR-1* antagonist AMO-1. The decisive step of presynaptic vesicle exocytosis is Ca^2+^-triggered fusion between synaptic vesicles and presynaptic membranes, which is mediated by the interaction of SNAREs and SM proteins containing SNAP-25, VAMP-2, syntaxin-1A and Munc-18 [7]. Here, we found that both SNAP-25 and syntaxin-1A protein expression levels were decreased. To clarify the molecular mechanism, we used three strategies, including the screening of the bioinformatics database, and in vitro antisense antagonist and miR-masking techniques, and found that *miR-1* directly regulates SNAP-25 expression but has no regulatory effect on syntaxin-1A protein expression. Interestingly, a previous study using SNAP-25-deficient neuron reported that the knockdown of SNAP-25 does not modify fusion pore opening or the rate of endocytosis to a degree that could alter Ca^2+^-triggered release and FM dye release kinetics significantly [40]. In the present study, we observed that *miR-1* gain of function could induce a decline in synaptic vesicle exocytosis, which was prevented by shielding the function of SNAP-25 via masking the *miR-1* binding site in the 3’UTR of the *Snap25* gene located at the site of 413 ~ 420 bp. All these studies implied that *miR-1* could induce the impairment of synaptic vesicle exocytosis by inhibiting the expression of SNAP-25 protein. In spite of the fact that SNAP-25 is part of the basic core fusion machinery, the role of SNAP-25 in excitatory- or inhibitory- neurotransmission is controversial [40–43]. Although we were not able to assess how *miR-1* or SNAP-25 affects glutamaterelease or aminobutyric acid neurotransmission, we believe that it is possible that *miR-1* or SNAP-25 play a role in both excitatory and inhibitory neurotransmission. However, this hypothesis needs to be studied further. In addition, vesicle release is a cascade response process involving a battery of proteins including synapsins, synaptotagmin-1, Munc-13, Munc-18, compelxins and SNAREs proteins. In this study, we focused on SNARE proteins. Whether the other vesicle-related proteins are also involved in MI- mediated presynaptic plasticity needs to be studied further.

Our previous study demonstrated that the increased *miR-1* in the hippocampi of cardiac-specific *miR-1-*overexpressing Tg mice was not due to endogenous biogenesis, but was associated with exosomes that mediated *miR-1* transport from the heart to the brain [18, 19]. Importantly, exosomal *miR-1* was increased in both the heart and the blood [34, 44]. Because the physiological process of exosomes as transporters is to release the transported substances into recipient cells by fusing with the recipient cell membrane and then becoming part of the cell membrane, the exosomal *miR-1* in the hippocampus might not represent the transported *miR-1* level from the heart. In this study, to evaluate the role of cardiac derived exosomes in regulating hippocampal *miR-1* levels, we performed an intraperitoneal injection of GW4869 to prevent exosome biogenesis in the heart. We found that GW4869 not only inhibited the expression of CD63 in the heart, a marker of exosomes [17], but also inhibited the increased *miR-1* level in both the blood and hippocampi of Tg mice and improved the decreased SNAP-25 expression and SV distribution at the active zone in the hippocampi of Tg mice. In addition, if *miR-1* can travel through blood exosomes, then this molecule may reach other brain areas, such as the frontal cortex and even the whole brain, most likely downregulating SNAP-25. This study focused on the hippocampus because the hippocampus is the main brain area associated with spatial memory, especially at the early stage of AD. MI is also considered a risk factor for the onset of AD and may be planned into the preclinical stage of AD. Our previous study demonstrated that *miR-1* Tg mice displayed an impairment of spatial memory. However, whether the overexpression of *miR-1* in the heart could also induce morphological or functional remodelling in other brain areas and what is the molecular mechanism is very interesting and needs to be clarified further.

## Conclusions

Taken together, our findings demonstrate that the overexpression of *miR-1* in the heart attenuated synaptic vesicle exocytosis in the hippocampus by posttranscriptionally regulating SNAP-25 through exosome transportation **(**Fig. 9**)**. This study improved our understanding of the relationship between cardiovascular disease and brain dysfunction at the miRNA levels.

## Abbreviations

AMO-1: anti-*miR-1* oligonucleotide fragment
CBH: chronic brain hypoperfusion
miRNAs: microRNAs
NRNs: neonatal rat hippocampal and cortical neurons
ODN: oligodeoxynucleotides
SNAP-25: synaptosome-associated protein 25
SNARE: soluble N-ethylmaleimide-sensitive factor attachment protein receptor
Tg: transgenic

## References

[1] Bartel DP (2004). MicroRNAs: genomics, biogenesis, mechanism, and function. Cell.

[2] Gibbings DJ, Ciaudo C, Erhardt M, Voinnet O (2009). Multivesicular bodies associate with components of miRNA effector complexes and modulate miRNA activity. Nat Cell Biol.

[3] Cortez MA, Bueso-Ramos C, Ferdin J, Lopez-Berestein G, Sood AK, Calin GA (2011). MicroRNAs in body fluids--the mix of hormones and biomarkers. Nat Rev Clin Oncol.

[4] Chen X, Liang H, Zhang J, Zen K, Zhang CY (2012). Secreted microRNAs: a new form of intercellular communication. Trends Cell Biol.

[5] Ai J, Zhang R, Li Y, Pu J, Lu Y, Jiao J, Li K, Yu B, Li Z, Wang R (2010). Circulating microRNA-1 as a potential novel biomarker for acute myocardial infarction. Biochem Biophys Res Commun.

[6] Allegra A, Alonci A, Campo S, Penna G, Petrungaro A, Gerace D, Musolino C (2012). Circulating microRNAs: new biomarkers in diagnosis, prognosis and treatment of cancer (review). Int J Oncol.

[7] Sudhof TC (2013). A molecular machine for neurotransmitter release: synaptotagmin and beyond. Nat Med.

[8] Gaffield MA, Betz WJ (2006). Imaging synaptic vesicle exocytosis and endocytosis with FM dyes. Nat Protoc.

[9] Vijayan M, Kumar S, Yin X, Zafer D, Chanana V, Cengiz P, Reddy PH (2018). Identification of novel circulatory microRNA signatures linked to patients with ischemic stroke. Hum Mol Genet.

[10] Redis RS, Calin S, Yang Y, You MJ, Calin GA (2012). Cell-to-cell miRNA transfer: from body homeostasis to therapy. Pharmacol Ther.

[11] Chen X, Liang H, Zhang J, Zen K, Zhang CY (2012). Horizontal transfer of microRNAs: molecular mechanisms and clinical applications. Protein Cell.

[12] Zernecke A, Bidzhekov K, Noels H, Shagdarsuren E, Gan L, Denecke B, Hristov M, Koppel T, Jahantigh MN, Lutgens E (2009). Delivery of microRNA-126 by apoptotic bodies induces CXCL12-dependent vascular protection. Sci Signal.

[13] Vickers KC, Palmisano BT, Shoucri BM, Shamburek RD, Remaley AT (2011). MicroRNAs are transported in plasma and delivered to recipient cells by high-density lipoproteins. Nat Cell Biol.

[14] Valadi H, Ekstrom K, Bossios A, Sjostrand M, Lee JJ, Lotvall JO (2007). Exosome-mediated transfer of mRNAs and microRNAs is a novel mechanism of genetic exchange between cells. Nat Cell Biol.

[15] Zhang Z, Li X, Sun W, Yue S, Yang J, Li J, Ma B, Wang J, Yang X, Pu M (2017). Loss of exosomal miR-320a from cancer-associated fibroblasts contributes to HCC proliferation and metastasis. Cancer Lett.

[16] Casadei L, Calore F, Creighton CJ, Guescini M, Batte K, Iwenofu OH, Zewdu A, Braggio DA, Bill KL, Fadda P (2017). Exosome-derived miR-25-3p and miR-92a-3p stimulate Liposarcoma progression. Cancer Res.

[17] Kosaka N, Iguchi H, Yoshioka Y, Takeshita F, Matsuki Y, Ochiya T (2010). Secretory mechanisms and intercellular transfer of microRNAs in living cells. J Biol Chem.

[18] Ma JC, Duan MJ, Sun LL, Yan ML, Liu T, Wang Q, Liu CD, Wang X, Kang XH, Pei SC (2015). Cardiac over-expression of microRNA-1 induces impairment of cognition in mice. Neuroscience.

[19] Sun LL, Duan MJ, Ma JC, Xu L, Mao M, Biddyut D, Wang Q, Yang C, Zhang S, Xu Y (2018). Myocardial infarction-induced hippocampal microtubule damage by cardiac originating microRNA-1 in mice. J Mol Cell Cardiol.

[20] Jellinger KA (2008). The pathology of "vascular dementia": a critical update. J Alzheimers Dis.

[21] de Toledo Ferraz Alves TC, Ferreira LK, Wajngarten M, Busatto GF (2010). Cardiac disorders as risk factors for Alzheimer's disease. J Alzheimers Dis.

[22] Monsuez JJ, Gesquiere-Dando A, Rivera S (2011). Cardiovascular prevention of cognitive decline. Cardiol Res Pract.

[23] Roberts RO, Knopman DS, Geda YE, Cha RH, Roger VL, Petersen RC (2010). Coronary heart disease is associated with non-amnestic mild cognitive impairment. Neurobiol Aging.

[24] de la Torre JC (2012). Cardiovascular risk factors promote brain hypoperfusion leading to cognitive decline and dementia. Cardiovasc Psychiatry Neurol.

[25] Vijayan M, Reddy PH (2016). Stroke, vascular dementia, and Alzheimer's disease: molecular links. J Alzheimers Dis.

[26] Ai J, Zhang R, Gao X, Niu HF, Wang N, Xu Y, Li Y, Ma N, Sun LH, Pan ZW, et al. Overexpression of microRNA-1 impairs cardiac contractile function by damaging sarcomere assembly. Cardiovasc Res. 2012;95:385-93. 10.1093/cvr/cvs196 22719074

[27] Yang B, Lin H, Xiao J, Lu Y, Luo X, Li B, Zhang Y, Xu C, Bai Y, Wang H (2007). The muscle-specific microRNA miR-1 regulates cardiac arrhythmogenic potential by targeting GJA1 and KCNJ2. Nat Med.

[28] Verstegen AM, Tagliatti E, Lignani G, Marte A, Stolero T, Atias M, Corradi A, Valtorta F, Gitler D, Onofri F (2014). Phosphorylation of synapsin I by cyclin-dependent kinase-5 sets the ratio between the resting and recycling pools of synaptic vesicles at hippocampal synapses. J Neurosci.

[29] Ai J, Sun LH, Che H, Zhang R, Zhang TZ, Wu WC, Su XL, Chen X, Yang G, Li K (2013). MicroRNA-195 protects against dementia induced by chronic brain hypoperfusion via its anti-amyloidogenic effect in rats. J Neurosci.

[30] Chen X, Jiang XM, Zhao LJ, Sun LL, Yan ML, Tian Y, Zhang S, Duan MJ, Zhao HM, Li WR (2017). MicroRNA-195 prevents dendritic degeneration and neuron death in rats following chronic brain hypoperfusion. Cell Death Dis.

[31] Che H, Yan Y, Kang XH, Guo F, Yan ML, Liu HL, Hou X, Liu T, Zong DK, Sun LL (2017). MicroRNA-27a promotes inefficient lysosomal clearance in the hippocampi of rats following chronic brain Hypoperfusion. Mol Neurobiol.

[32] Ivannikov MV, Sugimori M, Llinas RR (2013). Synaptic vesicle exocytosis in hippocampal synaptosomes correlates directly with total mitochondrial volume. J Mol Neurosci.

[33] Choi WY, Giraldez AJ, Schier AF (2007). Target protectors reveal dampening and balancing of nodal agonist and antagonist by miR-430. Science.

[34] Chistiakov DA, Orekhov AN, Bobryshev YV. Cardiac extracellular vesicles in Normal and infarcted heart. Int J Mol Sci. 2016;17:1-18 10.3390/ijms17010063 PMC4730308 26742038

[35] Cheng Y, Tan N, Yang J, Liu X, Cao X, He P, Dong X, Qin S, Zhang C (2010). A translational study of circulating cell-free microRNA-1 in acute myocardial infarction. Clin Sci (Lond).

[36] Liebetrau C, Mollmann H, Dorr O, Szardien S, Troidl C, Willmer M, Voss S, Gaede L, Rixe J, Rolf A (2013). Release kinetics of circulating muscle-enriched microRNAs in patients undergoing transcoronary ablation of septal hypertrophy. J Am Coll Cardiol.

[37] Dementia C (1977). Lancet.

[38] Jefferson AL (2010). Cardiac output as a potential risk factor for abnormal brain aging. J Alzheimers Dis.

[39] Ma JC, Duan MJ, Li KX, Biddyut D, Zhang S, Yan ML, Yang L, Jin Z, Zhao HM, Huang SY (2018). Knockdown of MicroRNA-1 in the Hippocampus ameliorates myocardial infarction induced impairment of long-term potentiation. Cell Physiol Biochem.

[40] Bronk P, Deak F, Wilson MC, Liu X, Sudhof TC, Kavalali ET (2007). Differential effects of SNAP-25 deletion on Ca2+ −dependent and Ca2+ −independent neurotransmission. J Neurophysiol.

[41] Tafoya LC, Mameli M, Miyashita T, Guzowski JF, Valenzuela CF, Wilson MC (2006). Expression and function of SNAP-25 as a universal SNARE component in GABAergic neurons. J Neurosci.

[42] Frassoni C, Inverardi F, Coco S, Ortino B, Grumelli C, Pozzi D, Verderio C, Matteoli M (2005). Analysis of SNAP-25 immunoreactivity in hippocampal inhibitory neurons during development in culture and in situ. Neuroscience.

[43] Verderio C, Pozzi D, Pravettoni E, Inverardi F, Schenk U, Coco S, Proux-Gillardeaux V, Galli T, Rossetto O, Frassoni C, Matteoli M (2004). SNAP-25 modulation of calcium dynamics underlies differences in GABAergic and glutamatergic responsiveness to depolarization. Neuron.

[44] Cheng Y, Wang X, Yang J, Duan X, Yao Y, Shi X, Chen Z, Fan Z, Liu X, Qin S (2012). A translational study of urine miRNAs in acute myocardial infarction. J Mol Cell Cardiol.

